# A 2-order additive fuzzy measure identification method based on hesitant fuzzy linguistic interaction degree and its application in credit assessment

**DOI:** 10.1038/s41598-024-58919-6

**Published:** 2024-04-14

**Authors:** Mu Zhang, Wen-jun Li, Cheng Cao

**Affiliations:** 1 School of Big Data Application and Economics, Guizhou University of Finance and Economics, Guiyang, 550025 China; 2 Guizhou Institution for Technology Innovation & Entrepreneurship Investment, Guizhou University of Finance and Economics, Guiyang, 550025 China

**Keywords:** Interactivity between attributes, Hesitant fuzzy linguistic term set, 2-order additive fuzzy measure, Choquet fuzzy integral, Multi-attribute decision making, Credit assessment, Engineering, Mathematics and computing

## Abstract

To reflect both fuzziness and hesitation in the evaluation of interactivity between attributes in the identification process of 2-order additive fuzzy measure, this work uses the hesitant fuzzy linguistic term set (HFLTS) to describe and depict the interactivity between attributes. Firstly, the interactivity between attributes is defined by the supermodular game theory. According to this definition, a linguistic term set is established to characterize the interactivity between attributes. Under the linguistic term set, the experts employ linguistic expressions generated by context-free grammar to qualitatively describe the interactivity between attributes. Secondly, through the conversion function, the linguistic expressions are transformed into the hesitant fuzzy linguistic term sets (HFLTSs). The individual evaluation results of all experts were further aggregated with the defined hesitant fuzzy linguistic weighted power average operator (HFLWPA). Thirdly, based on the standard Euclidean distance formula of the hesitant fuzzy linguistic elements (HFLEs), the hesitant fuzzy linguistic interaction degree (HFLID) between attributes is defined and calculated by constructing a piecewise function. As a result, a 2-order additive fuzzy measure identification method based on HFLID is proposed. Based on the proposed method, using the Choquet fuzzy integral as nonlinear integration operator, a multi-attribute decision making (MADM) process is then presented. Taking the credit assessment of the big data listed companies in China as an application example, the analysis results of application example prove the feasibility and effectiveness of the proposed method. This work successfully reflects both the fuzziness and hesitation in evaluating the interactivity between attributes in the identification process of 2-order additive fuzzy measure, enriches the theoretical framework of 2-order additive fuzzy measure, and expands the applicability and methodology of 2-order additive fuzzy measure in multi-attribute decision making.

## Introduction

In the process of multi-attribute decision making (MADM), due to the influence of the interaction among attributes, such as complementarity and repeatability, the classical weighted arithmetic mean method is often invalid^1^. To address this problem, to flexibly describe and depict any interaction among attributes, Sugeno^2^ proposed the concept of fuzzy measure and fuzzy integral. Since then, research on fuzzy measures had become increasingly rich, and gradually formed the theory of fuzzy measures^3,4^. However, in the practical application process, when there are *n* attributes, the general fuzzy measure usually needs to determine 2^*n*^ − 2 parameters^4,5^. This complexity greatly limits its practical application. Thereafter, to address the complexity of discrete fuzzy measures, Grabisch^6^ proposed the concept of *k*-order additive fuzzy measures, including usual additive measures and fuzzy measures. Every discrete fuzzy measure is a *k*-order additive fuzzy measure for a unique *k*. The *k*-order additive fuzzy measures cover all fuzzy measures with any complexity from classical additive measure (*k* = 1) to general fuzzy measure (*k* = *n*). Among them, the 2-order additive fuzzy measure only needs to determine $n(n + 1)/2$ parameters, involving the relative importance of attributes and the interaction between attributes, which solves the contradiction between complexity and performance ability well, so it has been widely used^7^.

However, due to the difficulty in explaining and understanding the interaction between attributes^8^ and the uncertainty of decision makers' cognition, decision makers often cannot give accurate quantitative evaluations of interactivity between attributes, which is often fuzzy and hesitant. The existing 2-order additive fuzzy measure identification methods mainly use subjective methods^9–16^ and objective methods^7,17–22^ to describe and depict the interactivity between attributes. Compared with the objective methods, the subjective methods are more explanatory, so they have been applied more widely. However, in the existing subjective methods, most methods cannot reflect the fuzziness of decision-making, while a few methods, although able to reflect the fuzziness of decision-making, cannot reflect the hesitation of decision-making. Therefore, it is urgent to construct an evaluation method for the interactivity between attributes that can reflect both fuzziness and hesitation.

In^23^, Rodríguez et al. introduced the concept of a hesitant fuzzy linguistic term set (HFLTS) to provide a linguistic and computational basis for increasing the flexibility and richness of linguistic elicitation based on the fuzzy linguistic approach and the use of context-free grammar to support the elicitation of linguistic information by experts in hesitant situations under qualitative settings. Hesitant fuzzy linguistic information is based on the linguistic expressions given by people; therefore, it is closer to people's thinking and cognition; and can flexibly and comprehensively reflect the real preferences of decision-makers^24^. HFLTS provides a new and powerful tool to characterize experts' qualitative decision information and has thus been successfully applied in the field of uncertain MADM^25–28^.

Based on this observation, to reflect both fuzziness and hesitation in the evaluation of interactivity between attributes, the present work uses the HFLTS to describe and depict the interactivity between attributes. As a result, a 2-order additive fuzzy measure identification method based on HFLID is proposed. The marginal contributions of this paper may be as follows: 1) The interactivity between attributes is defined by the supermodular game theory. According to this definition, a linguistic term set is established to characterize the interactivity between attributes. Under the linguistic term set, the experts employ the linguistic expressions generated by the context-free grammar to qualitatively describe the interactivity between attributes. (2) Through the conversion function, the linguistic expressions are transformed into HFLTSs. The individual evaluation results of all experts are further aggregated with the defined hesitant fuzzy linguistic weighted power average operator (HFLWPA). (3) Based on the standard Euclidean distance formula of the hesitant fuzzy linguistic elements (HFLEs), the hesitant fuzzy linguistic interaction degree (HFLID) between attributes is defined and calculated by constructing a piecewise function.

Based on the proposed method, using the Choquet fuzzy integral as a nonlinear integration operator^29,30^, a MADM process is further presented and applied to the credit assessment of big data enterprises. The remaining part of this paper is structured as follows: “Preparatory knowledge” introduces the preparatory knowledge employed in this study. “The proposed method” proposes the 2-order additive fuzzy measure identification method based on HFLID. “A MADM process based on the proposed method” presents the MADM process based on the proposed method. “Application Example” describes the application example analysis results. “Discussion” discusses the results obtained and “Conclusion” concludes this paper.

## Preparatory knowledge

This section introduces the related definitions of HFLTS, 2-order additive fuzzy measure and Choquet fuzzy integral reported in the literature. This is the basis of “The proposed method” and “A MADM process based on the proposed method”.

### Related definitions of HFLTS

#### Definition 1

^23,31^: Let $X = \left\{ {x_{1} ,x_{2} , \cdots ,x_{n} } \right\}$ be a universe of discourse, and $S = \left\{ {s_{\beta } \left| {\beta = - \tau , \cdots , - 1,0,1, \cdots ,\tau } \right.} \right\}$ be a linguistic term set. A hesitant fuzzy linguistic term set (HFLTS) in *X* is an object having the form

$$H_{S} = \left\{ { < x_{i} ,h_{S} (x_{i} ) > \left| {x_{i} \in X} \right.} \right\}$$

where $h_{S} (x_{i} )$ is the set of elements in *S*, called hesitant fuzzy linguistic element (HFLE), it can be expressed as $h_{S} (x_{i} ) = \left\{ {s_{{\varphi_{l} }} (x_{i} )\left| {s_{{\varphi_{l} }} (x_{i} ) \in S,l = 1,2, \cdots ,L} \right.} \right\}$, where $s_{{\varphi_{l} }} (x_{i} )$ is the φ*l*-th element in $h_{S} (x_{i} )$, *L* denotes the number of elements in $h_{S} (x_{i} )$.

#### Definition 2

^24,31^: Let $G_{H}$ be a context-free grammar, and $S = \left\{ {s_{\beta } \left| {\beta = - \tau , \cdots , - 1,0,1, \cdots ,\tau } \right.} \right\}$ be a linguistic term set. The elements of $G_{H} = (V_{N} ,V_{T} ,I,P)$ are defined as follows:

$V_{N}$ = {primary term, composite term, unary relation, binary relation, conjunction}; $V_{T}$ = {“less than”, “more than”, “at least”, “at most”, “between”, “and”, “$s_{ - \tau }$”, $\cdots$, “$s_{ - 1}$”, “$s_{0}$”, “$s_{1}$”, $\cdots$, “$s_{\tau }$”}; $I \in V_{N}$; $P$ = {$I$ refers to the primary term or composite term; the primary term refers to “$s_{ - \tau }$”, $\cdots$, “$s_{ - 1}$”, “$s_{0}$”, “$s_{1}$”, $\cdots$, “$s_{\tau }$”; the composite term refers to unary relation + primary term, or binary relation + primary term + conjunction + primary term; the unary relation refers to “less than” or “more than” or “at least” or “at most”; the binary relation refers to “between”; the conjunction refers to “and”}.

#### Definition 3

^24,31^: Let $S = \left\{ {s_{\beta } \left| {\beta = - \tau , \cdots , - 1,0,1, \cdots ,\tau } \right.} \right\}$ be a linguistic term set. Under the *S*, the language expression generated by $G_{H}$ is $ll \in S_{ll}$, where $S_{ll}$ is the set of all language expressions. Then the $S_{ll}$ can be transformed into the HFLTS by the transformation function $E_{{G_{H} }} :ll \to H_{S}$:

- (1) $$E_{{G_{H} }} (s_{t} ) = \left\{ {s_{t} \left| {s_{t} \in S} \right.} \right\}$$;
- (2) $$E_{{G_{H} }} ({\text{at most }}s_{m} ) = \left\{ {s_{t} \left| {s_{t} \in S,{\text{ and }}s_{t} \le s_{m} } \right.} \right\}$$;
- (3) $$E_{{G_{H} }} ({\text{less than }}s_{m} ) = \left\{ {s_{t} \left| {s_{t} \in S{\text{, and }}s_{t} < s_{m} } \right.} \right\}$$;
- (4) $$E_{{G_{H} }} ({\text{at least }}s_{m} ) = \left\{ {s_{t} \left| {s_{t} \in S{\text{, and }}s_{t} \ge s_{m} } \right.} \right\}$$;
- (5) $$E_{{G_{H} }} ({\text{more than }}s_{m} ) = \left\{ {s_{t} \left| {s_{t} \in S{\text{, and }}s_{t} > s_{m} } \right.} \right\}$$;
- (6) $$E_{{G_{H} }} ({\text{between }}s_{m} {\text{ and }}s_{n} ) = \left\{ {s_{t} \left| {s_{t} \in S{\text{, and }}s_{m} \le s_{t} \le s_{n} } \right.} \right\}$$.

#### Definition 4

^32^: Let $S = \left\{ {s_{\beta } \left| {\beta = - \tau , \cdots , - 1,0,1, \cdots ,\tau } \right.} \right\}$ be a linguistic term set, $h_{S} = \left\{ {s_{{\eta_{l} }} \left| {s_{{\eta_{l} }} \in S,l = 1,2, \cdots ,L} \right.} \right\}$ is a HFLE defined on *S*, then the mean value of $h_{S}$ is defined as

$$\mu (h_{S} ) = \frac{1}{L}\sum\limits_{l = 1}^{L} {\eta_{l} }$$

and the variance of $h_{S}$ is defined as

$$\nu (h_{S} ) = \frac{1}{L}\sum\limits_{l = 1}^{L} {(\eta_{l} - \mu (h_{S} ))^{2} }$$

Then, the binary relationship between $h_{S}^{i} = \left\{ {s_{{\eta_{l}^{i} }} \left| {s_{{\eta_{l}^{i} }} \in S,l = 1,2, \cdots ,L} \right.} \right\}$ and $h_{S}^{j} = \left\{ {s_{{\eta_{l}^{j} }} \left| {s_{{\eta_{l}^{j} }} \in S,l = 1,2, \cdots ,L} \right.} \right\}$ is defined as follows:

- (1) If $$\mu (h_{S}^{i} ) > \mu (h_{S}^{j} )$$, then $$h_{S}^{i} > h_{S}^{j}$$;
- (2) If $\mu (h_{S}^{i} ) = \mu (h_{S}^{j} )$, when $\nu (h_{S}^{i} ) = \nu (h_{S}^{j} )$, then $h_{S}^{i} = h_{S}^{j}$; when $\nu (h_{S}^{i} ) > \nu (h_{S}^{j} )$, then $h_{S}^{i} < h_{S}^{j}$; when $\nu (h_{S}^{i} ) < \nu (h_{S}^{j} )$, then $h_{S}^{i} > h_{S}^{j}$.

For convenience of calculation, referring to^33^, the $h_{S}$ with fewer elements can be expanded by adding element $s_{{\eta_{l} }} = \zeta s_{{\eta_{l} }}^{ + } \oplus (1 - \zeta )s_{{\eta_{l} }}^{ - }$ until meeting the need of calculation, where $s_{{\eta_{l} }}^{ + }$ and $s_{{\eta_{l} }}^{ - }$ is the largest and smallest element in $h_{S}$ respectively, and $\zeta \in [0,1]$ is the adjustment parameter. Without loss of generality, $\zeta = 0.5$ is usually taken.

#### Definition 5

^34^: Let $S = \left\{ {s_{\beta } \left| {\beta = - \tau , \cdots , - 1,0,1, \cdots ,\tau } \right.} \right\}$ be a linguistic term set, $h_{S}^{i} = \left\{ {s_{{\eta_{l}^{i} }} \left| {s_{{\eta_{l}^{i} }} \in S,l = 1,2, \cdots ,L} \right.} \right\}$ and $h_{S}^{j} = \left\{ {s_{{\eta_{l}^{j} }} \left| {s_{{\eta_{l}^{j} }} \in S,l = 1,2, \cdots ,L} \right.} \right\}$ are two HFLEs defined on *S*, if

1 $$d(h_{S}^{i} ,h_{S}^{j} ) = \sqrt {\frac{1}{L}\sum\limits_{l = 1}^{L} {\left( {\frac{{\eta_{l}^{i} - \eta_{l}^{j} }}{2\tau }} \right)^{2} } }$$

then $d(h_{S}^{i} ,h_{S}^{j} )$ is called the standard Euclidean distance between $h_{S}^{i}$ and $h_{S}^{j}$.

#### Definition 6

^34^: Let $S = \left\{ {s_{\beta } \left| {\beta = - \tau , \cdots , - 1,0,1, \cdots ,\tau } \right.} \right\}$ be a linguistic term set, $h_{S}^{1} ,h_{S}^{2} , \cdots ,h_{S}^{n}$ are *n* HFLEs defined on *S*. Let ${\text{HFLPA}}:\Theta^{n} \to \Theta$, if

2 $${\text{HFLPA}}(h_{S}^{1} ,h_{S}^{2} , \cdots ,h_{S}^{n} ) = \oplus_{i = 1}^{n} \frac{{1 + T(h_{S}^{i} )}}{{\sum\nolimits_{i = 1}^{n} {(1 + T(h_{S}^{i} ))} }}h_{S}^{i}$$

then HFLPA is called the hesitant fuzzy linguistic power average operator, where $T(h_{S}^{i} ) = \sum\nolimits_{j = 1,j \ne i}^{n} {\sup (h_{S}^{i} ,h_{S}^{j} } )$, the support function $\sup (h_{S}^{i} ,h_{S}^{j} )$ represents the support degree of $h_{S}^{i}$ and $h_{S}^{j}$, it satisfies the following three conditions:

- (1) $$\sup (h_{S}^{i} ,h_{S}^{j} ) \in [0,1]$$;
- (2) $$\sup (h_{S}^{i} ,h_{S}^{j} ) = \sup (h_{S}^{j} ,h_{S}^{i} )$$;
- (3) If $$d(h_{S}^{i} ,h_{S}^{j} ) < d(h_{S}^{s} ,h_{S}^{t} )$$, then $$\sup (h_{S}^{i} ,h_{S}^{j} ) > \sup (h_{S}^{s} ,h_{S}^{t} )$$.

### Related definitions of 2-order additive fuzzy measure and Choquet fuzzy integral

#### Definition 7

^2^: Let $X = \left\{ {x_{1} ,x_{2} , \cdots ,x_{n} } \right\}$ be a set of attributes, let $X^{ * } = \left\{ {1,2, \cdots ,n} \right\}$ be a set of subscripts of attributes. $P(X)$ is the power set of *X*, if the set function $g:P(X) \to [0,1]$ satisfies the following two conditions:

- (1) $$g(\emptyset ) = 0$$, $$g(X) = 1$$;
- (2) If $K \in P(X)$, $T \in P(X)$, $K \subseteq T$, then $g(K) \le g(T)$; then *g* is called a fuzzy measure on $P(X)$.

Grabisch^6^ proposed the *k*-order additive fuzzy measure based on pseudo-Boolean function and Möbius transformation. On this basis, the 2-order additive fuzzy measure is then defined as

3 $$g(K) = \sum\limits_{{i \in K^{ * } }} {m_{i} } + \sum\limits_{{\left\{ {i,j} \right\} \subset K^{ * } }} {m_{ij} } ,\;\forall K \subseteq X$$

where *m*_*i*_ is the Möbius transformation coefficient of $x_{i}$ ($i = 1,2, \cdots ,n$), which is an overall importance; *m*_*ij*_ is the Möbius transformation coefficient of $\left\{ {x_{i} ,x_{j} } \right\}$ ($i,j = 1,2, \cdots ,n$; $i \ne j$), which represents the extent of interaction between $x_{i}$ and $x_{j}$.

#### Definition 8

^13^: Let $X = \left\{ {x_{1} ,x_{2} , \cdots ,x_{n} } \right\}$ be a set of attributes, $W = \left\{ {w_{1} ,w_{2} , \cdots ,w_{n} } \right\}$ is the weight set of *X*, the Möbius transformation coefficients of $x_{i}$ and $\left\{ {x_{i} ,x_{j} } \right\}$ are respectively

4 $$\left\{ {\begin{array}{*{20}c} {m_{i} = {\raise0.7ex\hbox{${w_{i} }$} \!\mathord{\left/ {\vphantom {{w_{i} } P}}\right.\kern-0pt} \!\lower0.7ex\hbox{$P$}}} \\ {m_{ij} = {\raise0.7ex\hbox{${\xi_{ij} w_{i} w_{j} }$} \!\mathord{\left/ {\vphantom {{\xi_{ij} w_{i} w_{j} } P}}\right.\kern-0pt} \!\lower0.7ex\hbox{$P$}}} \\ \end{array} ,\;\;i,j = 1,2, \cdots ,n} \right.;i \ne j$$

where $P = \sum\limits_{{i \in X^{ * } }} {w_{i} } + \sum\limits_{{\left\{ {i,j} \right\} \subset X^{ * } }} {\xi_{ij} w_{i} w_{j} }$ is the sum of the importance of all $x_{i}$ and $\left\{ {x_{i} ,x_{j} } \right\}$, $\xi_{ij}$ is the interaction degree between $x_{i}$ and $x_{j}$, $\xi_{ij} \in [ - 1,1].$

#### Definition 9

^35^: Let *f* be a nonnegative function defined on *X*, *F* is a *σ*-algebra composed of subsets of *X* (when *X* is finite, *F* is the power set $P(X)$ of *X*), *g* is a fuzzy measure defined on *F*, then the Choquet fuzzy integral of function *f* on set *X* for fuzzy measure *g* is defined as

$$(c)\int {fdg = \int_{0}^{\infty } {g(F_{\alpha } )} } d\alpha$$

where $F_{\alpha } = \left\{ {x\left| {f(x) \ge \alpha ,x \in X} \right.} \right\}$, $\alpha \in [0,\infty ]$; $\int_{0}^{\infty } {g(F_{\alpha } )} d\alpha$ is the Riemann integral.

When *X* is a finite set, the elements in *X* are rearranged as $\left\{ {x_{(1)} ,x_{(2)} , \cdots ,x_{(n)} } \right\}$, which makes $f(x_{(1)} ) \le f(x_{(2)} ) \le \cdots \le f(x_{(n)} )$. Let $H{ = }(c)\int {fdg}$, then the Choquet fuzzy integral has the following simplified formula:

5 $$H = (c)\int {fdg} = \sum\limits_{i = 1}^{n} {\left[ {f(x_{(i)} ) - f(x_{(i - 1)} )} \right]} g(X_{(i)} )$$

where $$X_{(i)} = \left\{ {x_{(i)} ,x_{(i + 1)} , \cdots ,x_{(n)} } \right\}$$, $$(i) = (1),(2), \cdots ,(n)$$; $$f(x_{(0)} ) = 0$$.

## The proposed method

This section uses the HFLTS to describe and depict the interactivity between attributes, and then proposes a 2-order additive fuzzy measure identification method based on HFLID. In addition, the correctness of the proposed method is proved theoretically.

**Figure 1:**
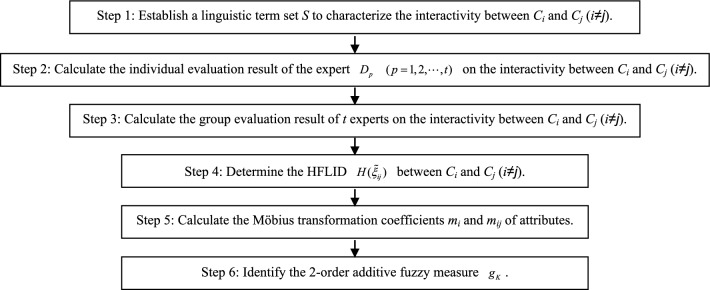
The identification process of 2-order additive fuzzy measure based on HFLID.

Let $A = (A_{1} ,A_{2} , \cdots ,A_{m} )$ be a finite set of alternatives, and $C = (C_{1} ,C_{2} , \cdots ,C_{n} )$ be a set of attributes to compare the alternatives, let $C^{ * } = \left\{ {1,2, \cdots ,n} \right\}$ be a set of subscripts of attributes. The weight vector of attributes is $W_{C} = (w_{1} ,w_{2} , \cdots ,w_{n} )$, where $w_{i} \in [0,1]$, and $\sum\nolimits_{i = 1}^{n} {w_{i} } = 1$. Let $D = (D_{1} ,D_{2} , \cdots ,D_{t} )$ be a set of experts, the weight vector of experts is $W_{D} = (w_{1} ,w_{2} , \cdots ,w_{t} )$, where $w_{p} \in [0,1]$, and $\sum\nolimits_{p = 1}^{t} {w_{p} } = 1$. Using the HFLTS, the identification process of 2-order additive fuzzy measure based on HFLID is shown in Fig. 1.

Step 1: Establish a linguistic term set *S* to characterize the interactivity between *C*_*i*_ and *C*_*j*_ (*i* ≠ *j*).

According to the supermodular game theory^36^, the interactivity between attributes is defined as follows:

### Definition 10

Let *C*_*i*_ and *C*_*j*_ (*i* ≠ *j*) have a partial order relation in the attribute set *C*, and the supremum $C_{i} \vee C_{j}$ and infimum $C_{i} \wedge C_{j}$ are in *C*, then *C* is called a sub-lattice^36^. Let *f* be a real-valued function defined on a sub-lattice *C*, $C \subseteq R^{n}$. For $\forall C_{i} ,C_{j} \in C$, when $f(C_{i} \vee C_{j} ) + f(C_{i} \wedge C_{j} ) > f(C_{i} ) + f(C_{j} )$, *f* is a supermodular function^36^, then there is complementarity between *C*_*i*_ and *C*_*j*_ (*i* ≠ *j*); when $f(C_{i} \vee C_{j} ) + f(C_{i} \wedge C_{j} ) < f(C_{i} ) + f(C_{j} )$, *f* is a submodular function^36^, then there is repeatability between *C*_*i*_ and *C*_*j*_ (*i* ≠ *j*); in particular, when $f(C_{i} \vee C_{j} ) + f(C_{i} \wedge C_{j} ) = f(C_{i} ) + f(C_{j} )$, then there is independence between *C*_*i*_ and *C*_*j*_ (*i* ≠ *j*).

According to Definition 10a linguistic term set $S = \left\{ {s_{\beta } \left| {\beta = - \tau , \cdots , - 1,0,1, \cdots ,\tau } \right.} \right\}$ is established to characterize the interactivity between *C*_*i*_ and *C*_*j*_ (*i* ≠ *j*), where $s_{1} ,s_{2} , \cdots ,s_{\tau }$ are the linguistic terms describing complementarity, the larger $\beta$ is, the stronger the complementarity is; $s_{{{ - }\tau }} ,s_{{{ - }\tau { + 1}}} , \cdots ,s_{{{ - }1}}$ are the linguistic terms describing repeatability, the smaller $\beta$ is, the stronger the repeatability is; $s_{0}$ is then a linguistic term describing independence, at this point, $\beta { = 0}$.

Step 2: Calculate the individual evaluation result of the expert $D_{p}$ ($p = 1,2, \cdots ,t$) on the interactivity between *C*_*i*_ and *C*_*j*_ (*i* ≠ *j*).

Under the linguistic term set *S*, according to Definition 10, every expert employs the linguistic expressions $ll$ generated by the context-free grammar $G_{H}$ (see Definition 2) to evaluate the interactivity between *C*_*i*_ and *C*_*j*_ (*i* ≠ *j*) ($C_{n}^{2}$ pairs in total). Through the transformation function $E_{{G_{H} }} :ll \to H_{S}^{ij}$ (see Definition 3), the linguistic expressions $ll$ are further transformed into the HLFTS $H_{S}^{ij}$.

Let $$H_{S}^{ij(p)}$$ be the HFLTS of the expert $$D_{p}$$$$(p = 1,2, \cdots ,t)$$, $$h_{S}^{ij(p)} = \left\{ {s_{{\eta_{l}^{ij(p)} }} \left| {s_{{\eta_{l}^{ij(p)} }} \in S,l = 1,2, \cdots ,L} \right.} \right\}$$ is the HFLE in $$H_{S}^{ij(p)}$$ ($$C_{n}^{2}$$ pairs in total), thus, the individual evaluation result $$h_{S}^{ij(p)}$$ of the expert $$D_{p}$$ ($$p = 1,2, \cdots ,t$$) on the interactivity between *C*_*i*_ and *C*_*j*_ (*i* ≠ *j*) is then given.

Step 3: Calculate the group evaluation result of *t* experts on the interactivity between *C*_*i*_ and *C*_*j*_ (*i* ≠ *j*).

Based on Definition 6, considering the weights of experts, the hesitant fuzzy linguistic weighted power average operator (HFLWPA) is defined as follows:

### Definition 11

Let $S = \left\{ {s_{\beta } \left| {\beta = - \tau , \cdots , - 1,0,1, \cdots ,\tau } \right.} \right\}$ be a linguistic term set, $h_{S}^{ij(1)} ,h_{S}^{ij(2)} , \cdots ,h_{S}^{ij(t)}$ are *t* HFLEs defined on *S*. The weight vector of *t* HFLEs is $(w_{1} ,w_{2} , \cdots ,w_{t} )$, where $w_{p} \in [0,1]$, and $\sum\nolimits_{p = 1}^{t} {w_{p} } = 1$. Let ${\text{HFLWPA}}:\Theta^{t} \to \Theta$, if

6 $${\text{HFLWPA}}(h_{S}^{ij(1)} ,h_{S}^{ij(2)} , \cdots ,h_{S}^{ij(t)} ) = \oplus_{p = 1}^{t} \frac{{w_{p} (1 + T(h_{S}^{ij(p)} ))}}{{\sum\nolimits_{p = 1}^{t} {w_{p} (1 + T(h_{S}^{ij(p)} ))} }}h_{S}^{ij(p)}$$

then HFLWPA is called the hesitant fuzzy linguistic weighted power average operator (when $w_{p} = 1/t$, HFLWPA degenerates to HFLPA^34^), where $T(h_{S}^{ij(p)} ) = \sum\nolimits_{q = 1,q \ne p}^{t} {\sup (h_{S}^{ij(p)} ,h_{S}^{ij(q)} } )$, the support function $\sup (h_{S}^{ij(p)} ,h_{S}^{ij(q)} )$ represents the support degree of $h_{S}^{ij(p)}$ and $h_{S}^{ij(q)}$, it satisfies the following three conditions:

- (1) $$\sup (h_{S}^{ij(p)} ,h_{S}^{ij(q)} ) \in [0,1]$$;
- (2) $$\sup (h_{S}^{ij(p)} ,h_{S}^{ij(q)} ) = \sup (h_{S}^{ij(q)} ,h_{S}^{ij(p)} )$$;
- (3) If $$d(h_{S}^{ij(p)} ,h_{S}^{ij(q)} ) < d(h_{S}^{ij(r)} ,h_{S}^{ij(s)} )$$, then $$\sup (h_{S}^{ij(p)} ,h_{S}^{ij(q)} ) > \sup (h_{S}^{ij(r)} ,h_{S}^{ij(s)} )$$.

In^37^, Yager defined different support functions, which lead to different degrees of support. In this paper, we take the support function as follows: $\sup (h_{S}^{ij(p)} ,h_{S}^{ij(q)} ) = 1 - d(h_{S}^{ij(p)} ,h_{S}^{ij(q)} )$, where $d(h_{S}^{ij(p)} ,h_{S}^{ij(q)} )$ is the standard Euclidean distance between $h_{S}^{ij(p)}$ and $h_{S}^{ij(q)}$.

According to Definition 4, after expanding those $h_{S}^{ij(p)}$ ($p = 1,2, \cdots ,t$) with fewer elements, using the HFLWPA to aggregate the individual evaluation results of *t* experts, the group evaluation result of *t* experts on the interactivity between *C*_*i*_ and *C*_*j*_ (*i* ≠ *j*) is then obtained as $h_{S}^{ij} = \left\{ {s_{{\eta_{l}^{ij} }} \left| {s_{{\eta_{l}^{ij} }} \in S,l = 1,2, \cdots ,L} \right.} \right\}$.

Step 4: Determine the HFLID $H(\tilde{\xi }_{ij} )$ between *C*_*i*_ and *C*_*j*_ (*i* ≠ *j*).

According to the established linguistic term set *S* (see step 1), by constructing a piecewise function based on Definition 5, the hesitant fuzzy linguistic interaction degree (HFLID) between attributes is defined as follows:

### Definition 12

Let $S = \left\{ {s_{\beta } \left| {\beta = - \tau , \cdots , - 1,0,1, \cdots ,\tau } \right.} \right\}$ be a linguistic term set characterizing the interactivity between *C*_*i*_ and *C*_*j*_ (*i* ≠ *j*), $h_{S}^{ij} = \left\{ {s_{{\eta_{l}^{ij} }} \left| {s_{{\eta_{l}^{ij} }} \in S,l = 1,2, \cdots ,L} \right.} \right\}$ is the group evaluation result of *t* experts on the interactivity between *C*_*i*_ and *C*_*j*_ (*i* ≠ *j*). Let $S^{ + } = \left\{ {s_{\beta } \left| {\beta = 0,1, \cdots ,\tau } \right.} \right\}$ be a subset of *S* ($S^{ + } \subset S$), when $s_{{\eta_{l}^{ij} }} \in S^{ + }$, $h_{S}^{ij} = h_{{S^{ + } }}^{ij}$; let $S^{ - } = \left\{ {s_{\beta } \left| {\beta = - \tau , \cdots , - 1,0} \right.} \right\}$ be another subset of *S* ($S^{ - } \subset S$), when $s_{{\eta_{l}^{ij} }} \in S^{ - }$, $h_{S}^{ij} = h_{{S^{ - } }}^{ij}$. Let $h_{S}^{0} = \left\{ {s_{0} \left| {s_{0} \in S} \right.} \right\}$, $h_{{S^{ + } }}^{0} = \left\{ {s_{0} \left| {s_{0} \in S^{ + } } \right.} \right\}$, $h_{{S^{ - } }}^{0} = \left\{ {s_{0} \left| {s_{0} \in S^{ - } } \right.} \right\}$, obviously, $h_{S}^{0} = h_{{S^{ + } }}^{0} = h_{{S^{ - } }}^{0}$. Based on Definition 5a piecewise function is constructed as

7 $$H(\tilde{\xi }_{ij} ) = \left\{ {\begin{array}{*{20}c} { + d(h_{{S^{ + } }}^{ij} ,h_{{S^{ + } }}^{0} ) = + \sqrt {\frac{1}{L}\sum\limits_{l = 1}^{L} {\left( {\frac{{\eta_{l}^{ij} }}{\tau }} \right)^{2} } } \quad \;s_{{\eta_{l}^{ij} }} \in S^{ + } ,l = 1,2, \cdots ,L} \\ { - d(h_{{S^{ - } }}^{ij} ,h_{{S^{ - } }}^{0} ) = - \sqrt {\frac{1}{L}\sum\limits_{l = 1}^{L} {\left( {\frac{{\eta_{l}^{ij} }}{\tau }} \right)^{2} } } \quad \;s_{{\eta_{l}^{ij} }} \in S^{ - } ,l = 1,2, \cdots ,L} \\ \end{array} } \right.\quad$$

where $H(\tilde{\xi }_{ij} )$ is called the hesitant fuzzy linguistic interaction degree (HFLID) between *C*_*i*_ and *C*_*j*_ (*i* ≠ *j*), obviously, $H(\tilde{\xi }_{ij} ) \in [ - 1,1]$. If there is complementarity between *C*_*i*_ and *C*_*j*_ (*i* ≠ *j*), then $H(\tilde{\xi }_{ij} ) > 0$, and the larger $H(\tilde{\xi }_{ij} )$ , the stronger complementarity. If there is repeatability between *C*_*i*_ and *C*_*j*_ (*i* ≠ *j*), then $H(\tilde{\xi }_{ij} ) < 0$, and the smaller $H(\tilde{\xi }_{ij} )$ , the stronger repeatability. If *C*_*i*_ and *C*_*j*_ (*i* ≠ *j*) are independent of each other, then $H(\tilde{\xi }_{ij} ) = 0$.

Therefore, according to Definition 12, the HFLID $H(\tilde{\xi }_{ij} )$ between *C*_*i*_ and *C*_*j*_ (*i* ≠ *j*) can be determined.

Step 5: Calculate the Möbius transformation coefficients *m*_*i*_ and *m*_*ij*_ of attributes.

According to the weight vector $W_{C} = (w_{1} ,w_{2} , \cdots ,w_{n} )$ of attributes and the HFLID $H(\tilde{\xi }_{ij} )$ between *C*_*i*_ and *C*_*j*_ (*i* ≠ *j*), the Möbius transformation coefficients *m*_*i*_ and *m*_*ij*_ of the attributes are calculated by using Eq. (4). The calculation formula is as follows:

8 $$\left\{ {\begin{array}{*{20}c} {m_{i} = {\raise0.7ex\hbox{${w_{i} }$} \!\mathord{\left/ {\vphantom {{w_{i} } P}}\right.\kern-0pt} \!\lower0.7ex\hbox{$P$}}} \\ {m_{ij} = {\raise0.7ex\hbox{${H(\tilde{\xi }_{ij} )w_{i} w_{j} }$} \!\mathord{\left/ {\vphantom {{H(\tilde{\xi }_{ij} )w_{i} w_{j} } P}}\right.\kern-0pt} \!\lower0.7ex\hbox{$P$}}} \\ \end{array} ,\;\;i,j = 1,2, \cdots ,n} \right.;i \ne j$$

where $P = \sum\limits_{{i \in C^{ * } }} {w_{i} } + \sum\limits_{{\left\{ {i,j} \right\} \subset C^{ * } }} {H(\tilde{\xi }_{ij} )w_{i} w_{j} }$ is the sum of the importance of all $C_{i}$ and $\left\{ {C_{i} ,C_{j} } \right\}$ (*i* ≠ *j*).

Step 6: Identify the 2-order additive fuzzy measure $g_{K}$.

According to the Möbius transformation coefficients *m*_*i*_ and *m*_*ij*_ of attributes, the 2-order additive fuzzy measure $g_{K}$ are calculated using Eq. (3). The calculation formula is as follows:

9 $$g_{K} = g(K) = \sum\limits_{{i \in K^{ * } }} {m_{i} } + \sum\limits_{{\left\{ {i,j} \right\} \subset K^{ * } }} {m_{ij} } ,\;\forall K \subseteq C$$

**Theorem 1**: The fuzzy measure identified by steps 1 to 6 is a 2-order additive fuzzy measure.

To prove that the fuzzy measure identified by steps 1 to 6 is a 2-order additive fuzzy measure, it is only necessary to prove that the calculated Möbius transformation coefficients satisfy the following constraints^6^:

- (1) $$m(\emptyset ) = 0$$;
- (2) $$m_{i} \ge 0$$, $$\forall i \in C^{ * }$$;
- (3) $$\sum\limits_{{i \in C^{ * } }} {m_{i} } + \sum\limits_{{\left\{ {i,j} \right\} \subset C^{ * } }} {m_{ij} } = 1$$;
- (4) $$m_{i} + \sum\limits_{{j \in K^{ * } \backslash \left\{ i \right\}}} {m_{ij} } \ge 0$$, $$\forall K \subset C$$.

### Proof:

(1) $$m(\emptyset ) = 0$$, obviously holds.

(2) Because $H(\tilde{\xi }_{ij} ) = H(\tilde{\xi }_{ji} )$, and $\sum\nolimits_{i = 1}^{n} {w_{i} } = 1$, *P* can be further written as

$$P = 1 + \frac{1}{2}\sum\limits_{i = 1}^{n} {\sum\limits_{j = 1,i \ne j}^{n} {H(\tilde{\xi }_{ij} )} } w_{i} w_{j} = 1 + \frac{1}{2}\sum\limits_{i = 1}^{n} {\left[ {w_{i} \sum\limits_{j = 1,i \ne j}^{n} {H(\tilde{\xi }_{ij} )} w_{j} } \right]}$$

Because $H(\tilde{\xi }_{ij} ) \in [ - 1,1]$, and $w_{j} \in \left[ {0,1} \right]$, we have

$$- w_{j} \le H(\tilde{\xi }_{ij} )w_{j} \le w_{j}$$, $$i \ne j$$.

Sum the two sides of the above inequality to *j*, we obtain

$$- (1 - w_{i} ) \le \sum\limits_{j = 1,i \ne j}^{n} {H(\tilde{\xi }_{ij} )w_{j} } \le 1 - w_{i}$$

Multiplying both sides of the above inequality by $w_{i}$, we obtain

$$- (w_{i} - w_{i}^{2} ) \le w_{i} \sum\limits_{j = 1,i \ne j}^{n} {H(\tilde{\xi }_{ij} )w_{j} } \le w_{i} - w_{i}^{2}$$

Sum the two sides of the above inequality to *i*, and the following inequality can be given

$$- (1 - \sum\limits_{i = 1}^{n} {w_{i}^{2} } ) \le \sum\limits_{i = 1}^{n} {\left[ {w_{i} \sum\limits_{j = 1,i \ne j}^{n} {H(\tilde{\xi }_{ij} )w_{j} } } \right]} \le 1 - \sum\limits_{i = 1}^{n} {w_{i}^{2} }$$

Therefore, we have

$$\frac{1}{2} + \frac{1}{2}\sum\limits_{i = 1}^{n} {w_{i}^{2} } \le 1 + \frac{1}{2}\sum\limits_{i = 1}^{n} {\left[ {w_{i} \sum\limits_{j = 1,i \ne j}^{n} {H(\tilde{\xi }_{ij} )w_{j} } } \right]} \le \frac{3}{2} - \frac{1}{2}\sum\limits_{i = 1}^{n} {w_{i}^{2} }$$

That is to say

$$0 < \frac{1}{2} + \frac{1}{2}\sum\limits_{i = 1}^{n} {w_{i}^{2} } \le P$$

Because $$P > 0$$, and $$w_{i} \ge 0$$, we can get $$m_{i} = {\raise0.7ex\hbox{${w_{i} }$} \!\mathord{\left/ {\vphantom {{w_{i} } P}}\right.\kern-0pt} \!\lower0.7ex\hbox{$P$}} \ge 0$$.

(3) $$\sum\limits_{{i \in C^{ * } }} {m_{i} } + \sum\limits_{{\left\{ {i,j} \right\} \subset C^{ * } }} {m_{ij} } = \sum\limits_{{i \in C^{ * } }} {{\raise0.7ex\hbox{${w_{i} }$} \!\mathord{\left/ {\vphantom {{w_{i} } P}}\right.\kern-0pt} \!\lower0.7ex\hbox{$P$}}} + \sum\limits_{{\left\{ {i,j} \right\} \subset C^{ * } }} {{\raise0.7ex\hbox{${H(\tilde{\xi }_{ij} )w_{i} w_{j} }$} \!\mathord{\left/ {\vphantom {{H(\tilde{\xi }_{ij} )w_{i} w_{j} } P}}\right.\kern-0pt} \!\lower0.7ex\hbox{$P$}}} = \frac{1}{P}\left[ {\sum\limits_{{i \in C^{ * } }} {w_{i} } + \sum\limits_{{\left\{ {i,j} \right\} \subset C^{ * } }} {H(\tilde{\xi }_{ij} )w_{i} w_{j} } } \right] = 1$$, obviously holds.

(4) $$m_{i} + \sum\limits_{{j \in K^{ * } \backslash \left\{ i \right\}}} {m_{ij} } = \frac{{w_{i} }}{P} + \sum\limits_{{j \in K^{ * } \backslash \left\{ i \right\}}} {\frac{{H(\tilde{\xi }_{ij} )w_{i} w_{j} }}{P}} = \frac{{w_{i} }}{P}\left[ {1 + \sum\limits_{{j \in K^{ * } \backslash \left\{ i \right\}}} {H(\tilde{\xi }_{ij} )w_{j} } } \right]$$.

Because $H(\tilde{\xi }_{ij} ) \in [ - 1,1]$, and $w_{j} \in \left[ {0,1} \right]$, we have $- (1 - w_{i} ) \le \sum\limits_{{j \in K^{ * } \backslash \left\{ i \right\}}} {H(\tilde{\xi }_{ij} )w_{j} } \le 1 - w_{i}$. Thus, the following inequality can be given

$$\frac{{w_{i} }}{P}\left[ {1 + \sum\limits_{{j \in K^{ * } \backslash \left\{ i \right\}}} {H(\tilde{\xi }_{ij} )w_{j} } } \right] \ge \frac{{w_{i} }}{P}\left[ {1 - (1 - w_{i} )} \right] \ge \frac{{w_{i}^{2} }}{P} \ge 0$$

Hence, we get $m_{i} + \sum\limits_{{j \in K^{ * } \backslash \left\{ i \right\}}} {m_{ij} } \ge 0$, $\forall K \subset C$. Q.E.D.

## A MADM process based on the proposed method

According to the 2-order additive fuzzy measure identification method based on HFLID, this section presents a MADM process.

Taking the Choquet fuzzy integral as a nonlinear integration operator, the MADM process based on the proposed method (see “The proposed method”) is as follows:

Step 1: Construct the normalized decision matrix $\tilde{X}$.

According to the types of attributes (including positive type, negative type, neutral type), the decision matrix $X$ is normalized to construct a normalized decision matrix $\tilde{X}$:

$$\tilde{X} = \left[ {\begin{array}{*{20}c} {\tilde{x}_{11} } & {\tilde{x}_{12} } & \cdots & {\tilde{x}_{1n} } \\ {\tilde{x}_{21} } & {\tilde{x}_{22} } & \cdots & {\tilde{x}_{2n} } \\ \vdots & \vdots & \ddots & \vdots \\ {\tilde{x}_{m1} } & {\tilde{x}_{m1} } & \cdots & {\tilde{x}_{mn} } \\ \end{array} } \right]_{m \times n}$$

where $\tilde{x}_{ji}$ is the normalized attribute value of alternative $A_{j}$ ($j = 1,2, \cdots ,m$) under attribute $C_{i}$ ($i = 1,2, \cdots ,n$).

Step 2: Determine the HFLID $H(\tilde{\xi }_{ij} )$ between *C*_*i*_ and *C*_*j*_ (*i* ≠ *j*).

Given the weight vector $W_{D}$ of experts, the HFLID $H(\tilde{\xi }_{ij} )$ between *C*_*i*_ and *C*_*j*_ (*i* ≠ *j*) can be determined by step 1–4 in “The proposed method”.

Step 3: Calculate the Möbius transformation coefficients *m*_*i*_ and *m*_*ij*_ of attributes.

Given the weight vector $W_{C}$ of attributes, according to the HFLID $H(\tilde{\xi }_{ij} )$ between *C*_*i*_ and *C*_*j*_ (*i* ≠ *j*), the Möbius transformation coefficients *m*_*i*_ and *m*_*ij*_ of the attributes are calculated by step 5 in “The proposed method”.

Step 4: Identify the 2-order additive fuzzy measure $g_{K}$.

According to the Möbius transformation coefficients *m*_*i*_ and *m*_*ij*_ of attributes, the 2-order additive fuzzy measure $g_{K}$ can be further identified by step 6 in “The proposed method”.

Step 5: Calculate the Choquet fuzzy integral values and the ranking of alternatives.

By reordering the normalized attribute value $\tilde{x}_{ji}$ ($i = 1,2, \cdots ,n$) of the alternative $A_{j}$ ($j = 1,2, \cdots ,m$) from small to large, the $\tilde{x}_{j(i)}$ can be obtained. Substituting the $\tilde{x}_{j(i)}$ and the 2-order additive fuzzy measure $g_{K}$ into Eq. (5), the Choquet fuzzy integral value $H_{j}$ of the alternative $A_{j}$ can be calculated. Simultaneously, the ranking of alternatives can be given, where the larger $H_{j}$, the better the alternative $A_{j}$.

## Application Example

This section uses the application example and data from^16^ to demonstrate the feasibility and effectiveness of the MADM process based on the proposed method.

### Credit assessment index system and sample data

Considering the characteristics of big data enterprises^38^, and following the principles of selecting indicators, such as scientificalness, objectivity, systematization, functionality, dynamics, relative independence, feasibility (or operability), comparability and so on, a credit assessment index system for big data enterprises was constructed, it included 6 primary indicators and 16 secondary indicators (see Table 1 in^16^), where the primary indicators included Debt Paying Ability (*C*_1_), Operational Capability (*C*_2_), Profitability (*C*_3_), Growth Capability (*C*_4_), Technological Innovation Capability (*C*_5_) and Industry Growth (*C*_6_).

**Table 1:** Individual evaluation results of interactivity.

Attributes* C*_*i*_ and *C*_*j*_ (*i* ≠ *j*)	Expert 1		Expert 2		Expert 3	
	$ll \in S_{ll}$	$h_{S}^{ij(1)}$	$ll \in S_{ll}$	$h_{S}^{ij(2)}$	$ll \in S_{ll}$	$h_{S}^{ij(3)}$
{*C*_1_, *C*_2_}	complementarity is relatively strong	{*s*_1_}	complementarity is relatively strong	{*s*_1_}	complementarity is between relatively strong and strong	{*s*_1_, *s*_2_}
{*C*_1_, *C*_3_}	complementarity is at least strong	{*s*_2_, *s*_3_, *s*_4_}	complementarity is strong	{*s*_2_}	complementarity is strong	{*s*_2_}
{*C*_1_, *C*_4_}	complementarity is between relatively strong and strong	{*s*_1_, *s*_2_}	complementarity is relatively strong	{*s*_1_}	complementarity is relatively strong	{*s*_1_}
{*C*_1_, *C*_5_}	complementarity is relatively strong	{*s*_1_}	complementarity is relatively strong	{*s*_1_}	complementarity is between relatively strong and strong	{*s*_1_, *s*_2_}
{*C*_1_, *C*_6_}	complementarity is relatively strong	{*s*_1_}	complementarity is between independence and relatively strong	{*s*_0_, *s*_1_}	complementarity is between independence and relatively strong	{*s*_0_, *s*_1_}
{*C*_2_, *C*_3_}	complementarity is between strong and very strong	{*s*_2_, *s*_3_}	complementarity is between strong and very strong	{*s*_2_, *s*_3_}	complementarity is strong	{*s*_2_}
{*C*_2_, *C*_4_}	complementarity is between relatively strong and strong	{*s*_1_, *s*_2_}	complementarity is between relatively strong and strong	{*s*_1_, *s*_2_}	complementarity is between independence and relatively strong	{*s*_0_, *s*_1_}
{*C*_2_, *C*_5_}	complementarity is relatively strong	{*s*_1_}	complementarity is between relatively strong and strong	{*s*_1_, *s*_2_}	complementarity is between relatively strong and strong	{*s*_1_, *s*_2_}
{*C*_2_, *C*_6_}	complementarity is relatively strong	{*s*_1_}	complementarity is between independence and relatively strong	{*s*_0_, *s*_1_}	complementarity is between independence and relatively strong	{*s*_0_, *s*_1_}
{*C*_3_, *C*_4_}	complementarity is between strong and very strong	{*s*_2_, *s*_3_}	complementarity is strong	{*s*_2_}	complementarity is strong	{*s*_2_}
{*C*_3_, *C*_5_}	complementarity is between relatively strong and strong	{*s*_1_, *s*_2_}	complementarity is strong	{*s*_2_}	complementarity is between relatively strong and strong	{*s*_1_, *s*_2_}
{*C*_3_, *C*_6_}	complementarity is between relatively strong and strong	{*s*_1_, *s*_2_}	complementarity is relatively strong	{*s*_1_}	complementarity is strong	{*s*_2_}
{*C*_4_, *C*_5_}	complementarity is between relatively strong and strong	{*s*_1_, *s*_2_}	complementarity is between relatively strong and strong	{*s*_1_, *s*_2_}	complementarity is strong	{*s*_2_}
{*C*_4_, *C*_6_}	repeatability is strong	{*s*_-2_}	repeatability is between strong and very strong	{*s*_-2_, *s*_-3_}	repeatability is strong	{*s*_-2_}
{*C*_5_, *C*_6_}	complementarity is relatively strong	{*s*_1_}	complementarity is between independence and relatively strong	{*s*_0_, *s*_1_}	complementarity is relatively strong	{*s*_1_}

We selected the big data listed companies in the Growth Enterprise Market (GEM) in China – Wangsu Science & Technology Co., Ltd. (300017), Beijing Lanxum Technology Co., Ltd. (300010), and Wuhan Tianyu Information Industry Co., Ltd. (300205) to form a set of alternatives, denoted by *A* = {*A*_1_, *A*_2_, *A*_3_}, where the alternative* A*_1_ and* A*_2_ belong to the software service industry, and the alternative *A*_3_ belongs to the electronic components industry. The sample data were the section data of 2016. We obtained a total of 48 original data (see Table 2 in^16^), where the original data of the Number of Invention Patent Applications Announced were from the Tian Yan Cha website, the original data of the Network Attention of Industry were from the Baidu Index website, and the rest of the original data were from the East Money website.

**Table 2:** Hesitant fuzzy linguistic interaction degrees between attributes.

Attributes* C*_*i*_ and *C*_*j*_ (*i* ≠ *j*)	Individual Evaluation Results of Interactivity (Expanded)			Group Evaluation Results of Interactivity	Hesitant Fuzzy Linguistic Interaction Degrees
	Expert 1	Expert 2	Expert 3		
{*C*_1_, *C*_2_}	{*s*_1_, *s*_1_, *s*_1_}	{*s*_1_, *s*_1_, *s*_1_}	{*s*_1_, *s*_1.5_, *s*_2_}	{*s*_1_, *s*_1.1471_, *s*_1.2941_}	0.2883
{*C*_1_, *C*_3_}	{*s*_2_, *s*_3_, *s*_4_}	{*s*_2_, *s*_2_, *s*_2_}	{*s*_2_, *s*_2_, *s*_2_}	{*s*_2_, *s*_2.3860_, *s*_2.7721_}	0.6017
{*C*_1_, *C*_4_}	{*s*_1_, *s*_1.5_, *s*_2_}	{*s*_1_, *s*_1_, *s*_1_}	{*s*_1_, *s*_1_, *s*_1_}	{*s*_1_, *s*_1.1966_, *s*_1.3933_}	0.3018
{*C*_1_, *C*_5_}	{*s*_1_, *s*_1_, *s*_1_}	{*s*_1_, *s*_1_, *s*_1_}	{*s*_1_, *s*_1.5_, *s*_2_}	{*s*_1_, *s*_1.1471_, *s*_1.2941_}	0.2883
{*C*_1_, *C*_6_}	{*s*_1_, *s*_1_, *s*_1_}	{*s*_0_, *s*_0.5_, *s*_1_}	{*s*_0_, *s*_0.5_, *s*_1_}	{*s*_0.3933_, *s*_0.6966_, *s*_1_}	0.1848
{*C*_2_, *C*_3_}	{*s*_2_, *s*_2.5_, *s*_3_}	{*s*_2_, *s*_2.5_, *s*_3_}	{*s*_2_, *s*_2_, *s*_2_}	{*s*_2_, *s*_2.3529_, *s*_2.7059_}	0.5926
{*C*_2_, *C*_4_}	{*s*_1_, *s*_1.5_, *s*_2_}	{*s*_1_, *s*_1.5_, *s*_2_}	{*s*_0_, *s*_0.5_, *s*_1_}	{*s*_0.7093_, *s*_1.2093_, *s*_1.7093_}	0.3191
{*C*_2_, *C*_5_}	{*s*_1_, *s*_1_, *s*_1_}	{*s*_1_, *s*_1.5_, *s*_2_}	{*s*_1_, *s*_1.5_, *s*_2_}	{*s*_1_, *s*_1.3034_, *s*_1.6067_}	0.3317
{*C*_2_, *C*_6_}	{*s*_1_, *s*_1_, *s*_1_}	{*s*_0_, *s*_0.5_, *s*_1_}	{*s*_0_, *s*_0.5_, *s*_1_}	{*s*_0.3933_, *s*_0.6966_, *s*_1_}	0.1848
{*C*_3_, *C*_4_}	{*s*_2_, *s*_1.5_, *s*_3_}	{*s*_2_, *s*_2_, *s*_2_}	{*s*_2_, *s*_2_, *s*_2_}	{*s*_2_, *s*_2.1966_, *s*_2.3933_}	0.5506
{*C*_3_, *C*_5_}	{*s*_1_, *s*_1.5_, *s*_2_}	{*s*_2_, *s*_2_, *s*_2_}	{*s*_1_, *s*_1.5_, *s*_2_}	{*s*_1.2941_, *s*_1.6471_, *s*_2_}	0.4180
{*C*_3_, *C*_6_}	{*s*_1_, *s*_1.5_, *s*_2_}	{*s*_1_, *s*_1_, *s*_1_}	{*s*_2_, *s*_2_, *s*_2_}	{*s*_1.2981_, *s*_1.5000_, *s*_1.7019_}	0.3773
{*C*_4_, *C*_5_}	{*s*_1_, *s*_1.5_, *s*_2_}	{*s*_1_, *s*_1.5_, *s*_2_}	{*s*_2_, *s*_2_, *s*_2_}	{*s*_1.2941_, *s*_1.6471_, *s*_2_}	0.4180
{*C*_4_, *C*_6_}	{*s*_-2_, *s*_-2_, *s*_-2_}	{*s*_-2_, *s*_-2.5_, *s*_-3_}	{*s*_-2_, *s*_-2_, *s*_-2_}	{*s*_-2_, *s*_-2.1471_, *s*_-2.2941_}	-0.5376
{*C*_5_, *C*_6_}	{*s*_1_, *s*_1_, *s*_1_}	{*s*_0_, *s*_0.5_, *s*_1_}	{*s*_1_, *s*_1_, *s*_1_}	{*s*_0.7059_, *s*_0.8529_, *s*_1_}	0.2153

### Process and results of credit assessment

Step 1: Construct the normalized decision matrix.

Using the algorithm given in^39^, based on the original data, the weight vector $W_{C}$ of attributes was calculated as^16^

$$W_{C} = (0.1702,0.1708,0.1810,0.1208,0.2261,0.1312)$$.

Combined with the algorithm given in^40^, based on the original data, the normalized decision matrix $\tilde{X}$ was further constructed as^16^

$$\tilde{X} = \left[ {\begin{array}{*{20}c} {0.4709} & {0.8976} & {\begin{array}{*{20}c} {0.9151} & {0.8192} & {0.8949} & {0.7789} \\ \end{array} } \\ {0.8662} & {0.6048} & {\begin{array}{*{20}c} {0.6148} & {0.9500} & {0.7786} & {0.7789} \\ \end{array} } \\ {0.8147} & {0.7671} & {\begin{array}{*{20}c} {0.1140} & {0.6054} & {0.2868} & {0.9156} \\ \end{array} } \\ \end{array} } \right]$$.

Step 2: Determine the HFLID $H(\tilde{\xi }_{ij} )$ between *C*_*i*_ and *C*_*j*_ (*i* ≠ *j*).

According to step 1 in “The proposed method”, a linguistic term set *S* was established to characterize the interactivity between *C*_*i*_ and *C*_*j*_ (*i* ≠ *j*), where *S* = {$s_{ - 4}$ = repeatability is extremely strong, $s_{ - 3}$ = repeatability is very strong, $s_{ - 2}$ = repeatability is strong, $s_{ - 1}$ = repeatability is relatively strong, $s_{0}$ = independence, $s_{1}$ = complementarity is relatively strong, $s_{2}$ = complementarity is strong, $s_{3}$ = complementarity is very strong, $s_{4}$ = complementarity is extremely strong }.

In this paper, three experts were invited to analyze the six attributes in pairs respectively. According to step 2 in “The proposed method”, under the linguistic term set *S*, according to Definition 10, every expert employed the linguistic expressions $ll$ generated by the context-free grammar $G_{H}$ to evaluate the interactivity between* C*_*i*_ and *C*_*j*_ (*i* ≠ *j*) ($C_{6}^{2}$ pairs in total). Through the transformation function $E_{{G_{H} }} :ll \to H_{S}^{ij}$, the linguistic expressions $ll$ were further transformed into the HLFTS $H_{S}^{ij}$. Thus, the individual evaluation results $h_{S}^{ij(p)}$ $(p = 1,2,3)$ of three experts on the interactivity between *C*_*i*_ and *C*_*j*_ (*i* ≠ *j*) were then given, as shown in Table 1.

Adopting the cycle mutual evaluation method^41^, the weight vector of experts was calculated as $W_{D} = (0.3976,0.3012,0.3012)$ (see Appendix A in^16^ for full calculation principle and process).

According to step 3 in “The proposed method”, after expanding those $h_{S}^{ij(p)}$ $(p = 1,2,3)$ with fewer elements (see Table 2), using the HFLWPA to aggregate the individual evaluation results of three experts, the group evaluation result of three experts on the interactivity between *C*_*i*_ and *C*_*j*_ (*i* ≠ *j*) was then obtained (see Table 2). According to step 4 in “The proposed method”, with Eq. (7), the HFLID $H(\tilde{\xi }_{ij} )$ between *C*_*i*_ and *C*_*j*_ (*i* ≠ *j*) was calculated, as shown in Table 2.

Taking $H(\tilde{\xi }_{12} )$ as an example, its calculation process was as follows:

(1) Using Eq. (1), the standard Euclidean distance among $h_{S}^{ij(p)}$ $(p = 1,2,3)$ was calculated as

$$d(h_{S}^{12(1)} ,h_{S}^{12(2)} ) = 0$$, $$d(h_{S}^{12(1)} ,h_{S}^{12(3)} ) = 0.0807$$, $$d(h_{S}^{12(2)} ,h_{S}^{12(3)} ) = 0.0807$$.

According to Definition 11, their corresponding support degree was also obtained as

$$\sup (h_{S}^{12(1)} ,h_{S}^{12(2)} ) = 1$$, $$\sup (h_{S}^{12(1)} ,h_{S}^{12(3)} ) = 0.9193$$, $$\sup (h_{S}^{12(2)} ,h_{S}^{12(3)} ) = 0.9193$$.

Thus, we can get

$$T(h_{S}^{12(1)} ) = 1.9193$$, $$T(h_{S}^{12(2)} ) = 1.9193$$, $$T(h_{S}^{12(3)} ) = 1.8386$$.

(2) Using Eq. (6), the group evaluation result of three experts on the interactivity between *C*_1_ and *C*_2_ was calculated as

$$h_{S}^{12} = \left\{ {s_{1} ,s_{1.1471} ,s_{1.2941} } \right\}$$.

(3) With Eq. (7), the HFLID $H(\tilde{\xi }_{12} )$ between *C*_1_ and *C*_2_ was then calculated as

$$H(\tilde{\xi }_{12} ) = d(h_{{S^{ + } }}^{12} ,h_{{S^{ + } }}^{0} ) = \sqrt {\frac{1}{3}\left( {\left( \frac{1}{4} \right)^{2} + \left( {\frac{1.1471}{4}} \right)^{2} + \left( {\frac{1.2941}{4}} \right)^{2} } \right)} = 0.2883$$.

Step 3: Calculate the Möbius transformation coefficients *m*_*i*_ and *m*_*ij*_ of attributes.

Based on the weight vector $W_{C}$ of attributes and Table 2, using Eq. (8), the Möbius transformation coefficients *m*_*i*_ and *m*_*ij*_ of attributes were calculated, as shown in Table 3, where $P = 1.1378$. Taking $m_{12}$ as an example, we had $m_{12} = (0.2883 \times 0.1702 \times 0.1708)/1.1378 = 0.0074$.

**Table 3:** Calculation results of Möbius transformation coefficients.

Möbius Transformation Coefficients	Coefficient Values	Möbius Transformation Coefficients	Coefficient Values	Möbius Transformation Coefficients	Coefficient Values
*m*_1_	0.1496	*m*_13_	0.0163	*m*_26_	0.0037
*m*_2_	0.1501	*m*_14_	0.0055	*m*_34_	0.0106
*m*_3_	0.1591	*m*_15_	0.0098	*m*_35_	0.0150
*m*_4_	0.1062	*m*_16_	0.0036	*m*_36_	0.0079
*m*_5_	0.1987	*m*_23_	0.0161	*m*_45_	0.0100
*m*_6_	0.1153	*m*_24_	0.0058	*m*_46_	-0.0075
*m*_12_	0.0074	*m*_25_	0.0113	*m*_56_	0.0056

Step 4: Identify the 2-order additive fuzzy measure $g_{K}$.

Based on Table 3, using Eq. (9), the 2-order additive fuzzy measure $g_{K}$ was calculated, as shown in Table 4. Taking $g_{{\left\{ {1,2} \right\}}}$ as an example, we had $g_{{\left\{ {1,2} \right\}}} = 0.1496 + 0.1501 + 0.0074 = 0.3071$.

**Table 4:** Calculation results of 2-order additive fuzzy measures.

*K*	*g*_*K*_	*K*	*g*_*K*_	*K*	*g*_*K*_	*K*	*g*_*K*_
{ø}	0.0000	{3, 4}	0.2758	{2, 3, 4}	0.4478	{1, 3, 4, 5}	0.6807
{1}	0.1496	{3, 5}	0.3728	{2, 3, 5}	0.5503	{1, 3, 4, 6}	0.5665
{2}	0.1501	{3, 6}	0.2823	{2, 3, 6}	0.4522	{1, 3, 5, 6}	0.6809
{3}	0.1591	{4, 5}	0.3149	{2, 4, 5}	0.4821	{1, 4, 5, 6}	0.5968
{4}	0.1062	{4, 6}	0.2140	{2, 4, 6}	0.3736	{2, 3, 4, 5}	0.6829
{5}	0.1987	{5, 6}	0.3196	{2, 5, 6}	0.4847	{2, 3, 4, 6}	0.5672
{6}	0.1153	{1, 2, 3}	0.4985	{3, 4, 5}	0.4996	{2, 3, 5, 6}	0.6828
{1, 2}	0.3071	{1, 2, 4}	0.4245	{3, 4, 6}	0.3915	{2, 4, 5, 6}	0.5992
{1, 3}	0.3250	{1, 2, 5}	0.5268	{3, 5, 6}	0.5016	{3, 4, 5, 6}	0.6209
{1, 4}	0.2612	{1, 2, 6}	0.4297	{4, 5, 6}	0.4284	{1, 2, 3, 4, 5}	0.8713
{1, 5}	0.3581	{1, 3, 4}	0.4472	{1, 2, 3, 4}	0.6265	{1, 2, 3, 4, 6}	0.7496
{1, 6}	0.2685	{1, 3, 5}	0.5485	{1, 2, 3, 5}	0.7333	{1, 2, 3, 5, 6}	0.8694
{2, 3}	0.3253	{1, 3, 6}	0.4518	{1, 2, 3, 6}	0.6291	{1, 2, 4, 5, 6}	0.7750
{2, 4}	0.2621	{1, 4, 5}	0.4797	{1, 2, 4, 5}	0.6542	{1, 3, 4, 5, 6}	0.8056
{2, 5}	0.3601	{1, 4, 6}	0.3727	{1, 2, 4, 6}	0.5396	{2, 3, 4, 5, 6}	0.8079
{2, 6}	0.2691	{1, 5, 6}	0.4826	{1, 2, 5, 6}	0.6551	{1, 2, 3, 4, 5, 6}	1.0000

Step 5: Calculate the Choquet fuzzy integral values and the ranking of alternatives.

Take the alternative $A_{1}$ as an example.

According to step 3 in “A MADM process based on the proposed method”, reordering the normalized attribute value $\tilde{x}_{1i}$ ($i = 1,2, \cdots ,6$) of alternative $A_{1}$ from small to large, we can get $\tilde{x}_{11} < \tilde{x}_{16} < \tilde{x}_{14} < \tilde{x}_{15} < \tilde{x}_{12} < \tilde{x}_{13}$, which can be denoted by

$$\tilde{x}_{1(1)} < \tilde{x}_{1(2)} < \tilde{x}_{1(3)} < \tilde{x}_{1(4)} < \tilde{x}_{1(5)} < \tilde{x}_{1(6)}$$.

Substituting the $\tilde{x}_{1(i)}$ and the 2-order additive fuzzy measure $g_{K}$ into Eq. (5), the Choquet fuzzy integral value of the alternative $A_{1}$ was calculated as

$$H_{1} = (0.4709 - 0.0000) \times 1.0000 + (0.7789 - 0.4709) \times 0.8079 + (0.8192 - 0.7789) \times 0.6829$$

$$+ (0.8949 - 0.8192) \times 0.5503 + (0.8976 - 0.8949) \times 0.3253 + (0.9151 - 0.8976) \times 0.1591 = 0.7926$$.

Similarly, we can also obtain $H_{2} = 0.7643$ and $H_{3} = 0.5138$.

Since $H_{1} > H_{2} > H_{3}$, then the ranking of alternatives was $A_{1} \succ A_{2} \succ A_{3}$.

That is to say, the credit status of the alternative $A_{1}$ was relatively good, and the credit status of the alternative $A_{3}$ was relatively poor.

### Comparative analysis

For comparison, we invited the above three experts to use the scoring method^13^, the diamond pairwise comparisons (DPC) method^9^ and the intuitionistic fuzzy sets (IFSs) method^16^ to determine the interaction degrees between attributes, respectively. Given the weight vector $W_{D} = (0.3976,0.3012,0.3012)$ of experts, the scoring interaction degrees^13^, the DPC interaction degrees^9^ and the intuitionistic fuzzy interaction degrees^16^ between attributes were further obtained, as shown in Table 5.

**Table 5:** The interaction degrees between attributes.

Attributes* C*_*i*_ and *C*_*j*_ (*i* ≠ *j*)	Scoring Interaction Degrees	DPC Interaction Degrees	Intuitionistic Fuzzy Interaction Degrees
{*C*_1_, *C*_2_}	0.2301	0.2801	0.2851
{*C*_1_, *C*_3_}	0.4205	0.4705	0.5815
{*C*_1_, *C*_4_}	0.2301	0.2801	0.2851
{*C*_1_, *C*_5_}	0.2301	0.2801	0.2851
{*C*_1_, *C*_6_}	0.1398	0.1747	0.1503
{*C*_2_, *C*_3_}	0.4205	0.4705	0.5815
{*C*_2_, *C*_4_}	0.2301	0.2801	0.2851
{*C*_2_, *C*_5_}	0.2301	0.2801	0.2851
{*C*_2_, *C*_6_}	0.1398	0.1747	0.1503
{*C*_3_, *C*_4_}	0.3602	0.4102	0.5206
{*C*_3_, *C*_5_}	0.2602	0.3102	0.3611
{*C*_3_, *C*_6_}	0.2602	0.3102	0.3611
{*C*_4_, *C*_5_}	0.2602	0.3102	0.3611
{*C*_4_, *C*_6_}	-0.4205	-0.4705	-0.5659
{*C*_5_, *C*_6_}	0.1398	0.1747	0.1503

Furthermore, we replaced the HFLIDs (see Table 2) in step 2 of “Process and results of credit assessment” with the scoring interaction degrees (see Table 5). Ceteris paribus, the Choquet fuzzy integral values of alternatives were calculated as

$$H^{\prime}_{1} = 0.7946$$, $$H^{\prime}_{2} = 0.7686$$, $$H^{\prime}_{3} = 0.5244$$.

Since $H^{\prime}_{1} > H^{\prime}_{2} > H^{\prime}_{3}$, then the ranking of alternatives was $A_{1} \succ A_{2} \succ A_{3}$.

Similarly, we also replaced the HFLIDs (see Table 2) in step 2 of “Process and results of credit assessment" with the DPC interaction degrees (see Table 5). Ceteris paribus, the Choquet fuzzy integral values of alternatives were calculated as

$$H^{\prime\prime}_{1} = 0.7931$$, $$H^{\prime\prime}_{2} = 0.7670$$, $$H^{\prime\prime}_{3} = 0.5211$$.

Since $H^{\prime\prime}_{1} > H^{\prime\prime}_{2} > H^{\prime\prime}_{3}$, then the ranking of alternatives was $A_{1} \succ A_{2} \succ A_{3}$.

In addition, we also replaced the HFLIDs (see Table 2) in step 2 of “Process and results of credit assessment” with the intuitionistic fuzzy interaction degrees (see Table 5). Ceteris paribus, the Choquet fuzzy integral values of alternatives were calculated as

$$H^{\prime\prime\prime}_{1} = 0.7927$$, $$H^{\prime\prime\prime}_{2} = 0.7652$$, $$H^{\prime\prime\prime}_{3} = 0.5157$$.

Since $H^{\prime\prime\prime}_{1} > H^{\prime\prime\prime}_{2} > H^{\prime\prime\prime}_{3}$, then the ranking of alternatives was $A_{1} \succ A_{2} \succ A_{3}$.

For simplicity, we referred to the MADM process based on the HFLIDs as Method 1 (our method), referred to the MADM process based on the scoring interaction degrees as Method 2, referred to the MADM process based on the DPC interaction degrees as Method 3, and referred to the MADM process based on the intuitionistic fuzzy interaction degrees as Method 4. The Choquet fuzzy integral values of alternatives of Method 1, Method 2, Method 3 and Method 4 were shown in Table 6.

**Table 6:** The Choquet fuzzy integral values of alternatives and discrimination.

Method	Choquet fuzzy integral values of *A*_1_	Choquet fuzzy integral values of *A*_2_	Choquet fuzzy integral values of *A*_3_	Discrimination
Method 1	0.7926	0.7643	0.5138	0.7153
Method 2	0.7946	0.7686	0.5244	0.6903
Method 3	0.7931	0.7670	0.5211	0.6965
Method 4	0.7927	0.7652	0.5157	0.7102

We further investigated the discrimination of Method 1 for alternatives, as well as Method 2, Method 3 and Method 4. Adopting the algorithm of discrimination given in ^42^ (see Appendix A for full calculation principle), the discrimination of Method 1 for alternatives was calculated as

$$\rho { = }\frac{0.7926 - 0.7643}{{0.7926}} + \frac{0.7926 - 0.5138}{{0.7926}} + \frac{0.7643 - 0.5138}{{0.7643}} = 0.7153$$.

Similarly, the discrimination of Method 2 for alternatives was calculated as

$$\rho^{\prime}{ = }\frac{0.7946 - 0.7686}{{0.7946}} + \frac{0.7946 - 0.5244}{{0.7946}} + \frac{0.7686 - 0.5244}{{0.7686}} = 0.6903$$.

Similarly, the discrimination of Method 3 for alternatives was calculated as

$$\rho^{\prime\prime} = \frac{0.7931 - 0.7670}{{0.7931}} + \frac{0.7931 - 0.5211}{{0.7931}} + \frac{0.7670 - 0.5211}{{0.7670}} = 0.6965$$.

Similarly, the discrimination of Method 4 for alternatives was calculated as

$$\rho^{\prime\prime\prime} = \frac{0.7927 - 0.7652}{{0.7927}} + \frac{0.7927 - 0.5157}{{0.7927}} + \frac{0.7652 - 0.5157}{{0.7652}} = 0.7102$$.

The discrimination of Method 1, Method 2, Method 3 and Method 4 for alternatives was shown in Table 6.

As shown in Table 6, although the ranking results of Method 2, Method 3 and Method 4 are consistent with that of Method 1, the discrimination of Method 1 for alternatives is higher than that of Method 2, Method 3 and Method 4, which means that the decision-making effect of Method 1 is better than that of Method 2, Method 3 and Method 4.

## Discussion

From the results and analysis of the previous section, we observed that Method 1 was able to obtain the higher discrimination value than Method 2, Method 3 and Method 4 (Method 1 was 0.7153, Method 2 was 0.6903, Method 3 was 0.6965 and Method 4 was 0.7102), and the slightly lower Choquet fuzzy integral mean value than Method 2, Method 3 and Method 4 (Method 1 was 0.6902, Method 2 was 0.6959, Method 3 was 0.6938 and Method 4 was 0.6912), where the Choquet fuzzy integral mean values of the four methods are calculated as follows:

$$\overline{H} = \frac{0.7926 + 0.7643 + 0.5138}{3} = 0.6902$$; $$\overline{H^{\prime}} = \frac{0.7946 + 0.7686 + 0.5244}{3} = 0.6959$$; $$\overline{H^{\prime\prime}} = \frac{0.7931 + 0.7670 + 0.5211}{3} = 0.6938$$; $$\overline{H}^{\prime\prime\prime} = \frac{0.7927 + 0.7652 + 0.5157}{3} = 0.6912$$.

where $\overline{H}$, $\overline{H^{\prime}}$, $\overline{H^{\prime\prime}}$ and $\overline{H}^{\prime\prime\prime}$ represents the mean value of $H$, $H^{\prime}$, $H^{\prime\prime}$ and $H^{\prime\prime\prime}$, respectively.

Compared with Method 2, Method 3 and Method 4, Method 1 can obtain the higher discrimination value. Since the variance of interaction degrees determined by Method 1 (its value was equal to 0.0673) was higher than that of Method 2 (its value was equal to 0.0355), Method 3 (its value was equal to 0.0449) and Method 4 (its value was equal to 0.0684). According to Eq. (4), the variances of *m*_*i*_ and *m*_*ij*_ increase with the increase of the variance of interaction degrees. From Eq. (3) and Eq. (5), we can further see that the variance of $g(K)$ and the variance of *H* also increase correspondingly. Thus, according to the algorithm of discrimination ^42^, the discrimination value becomes larger. This transmission mechanism is as follows:

$$\sigma_{{\xi_{ij} }}^{2} \uparrow \Rightarrow \sigma_{{m_{i} }}^{2} ,\sigma_{{m_{ij} }}^{2} \uparrow \Rightarrow \sigma_{g(K)}^{2} \uparrow \Rightarrow \sigma_{H}^{2} \uparrow \Rightarrow \rho \uparrow$$

where $\sigma_{{\xi_{ij} }}^{2}$, $\sigma_{{m_{i} }}^{2}$, $\sigma_{{m_{ij} }}^{2}$, $\sigma_{g(K)}^{2}$ and $\sigma_{H}^{2}$ represents the variance of $\xi_{ij}$, $m_{i}$, $m_{ij}$, $g(K)$ and $H$, respectively.

Compared with Method 2, Method 3 and Method 4, Method 1 can obtain the slightly lower Choquet fuzzy integral mean value. Since the average value of interaction degrees determined by Method 1 (its value was equal to 0.3023) was higher than that of Method 2 (its value was equal to 0.2087), Method 3 (its value was equal to 0.2490) and Method 4 (its value was equal to 0.2718). According to Eq. (4), when the average value of interaction degrees increases, the mean value of* P* increases, meanwhile, the mean value of *m*_*i*_ decreases and the mean value of* m*_*ij*_ increases. However, because $\left| {m_{i} } \right| \gg \left| {m_{ij} } \right|$, from Eq. (3), we can further see that the mean value of $g(K)$ also decreases correspondingly. Thus, according to Eq. (5), the mean value of *H* becomes smaller. This transmission mechanism is as follows:

$$\overline{\xi }_{ij} \uparrow \Rightarrow \overline{P} \uparrow \Rightarrow \overline{m}_{i} \Downarrow ,\overline{m}_{ij} \uparrow \Rightarrow \overline{g}(K) \downarrow \Rightarrow \overline{H} \downarrow$$

where $\overline{\xi }_{ij}$, $\overline{P}$, $\overline{m}_{i}$, $\overline{m}_{ij}$ and $\overline{g}(K)$ represents the average value of $\xi_{ij}$, $P$, $m_{i}$, $m_{ij}$ and $g(K)$, respectively.

Both the variance and the average value of interaction degrees determined by Method 1 were higher than that of Method 2, Method 3 and Method 4, the fundamental reasons are as follows: 1) The Method 1 defines the interactivity between attributes by using the supermodular game theory, so that the interaction between attributes is easier to explain and understand. According to this definition, the linguistic term set *S* is established to characterize the interactivity between attributes. The experts then employ the linguistic expressions generated by the context-free grammar to qualitatively describe the interactivity between attributes under the linguistic term set *S*, which makes the evaluation of the interactivity between attributes closer to reality. 2) In Method 1, through the conversion function, the linguistic expressions are transformed into the HFLTSs. The individual evaluation results of all experts are further aggregated by using the defined HFLWPA. The HFLID between attributes is then defined and calculated, thereby better preserving, and characterizing the experts’ evaluation information. 3) In Method 2, the experts use scoring method to determine the interaction degree between attributes according to experience and preference. The Method 3 uses diamond diagram to help decision makers intuitively determine the relative importance and interaction coefficient between attributes. These two methods lack both the theoretical definition of the interactivity between attributes and the fuzziness and hesitation of decision-making. In addition, the Method 4 uses the intuitionistic fuzzy sets (IFSs) to describe and depict the interactivity between attributes, however, the IFSs cannot reflect the hesitation of expert decision-making.

## Conclusion

The marginal contributions of this paper may be as follows: 1) The interactivity between attributes is defined by the supermodular game theory. According to this definition, a linguistic term set is established to characterize the interactivity between attributes. Under the linguistic term set, the experts employ the linguistic expressions generated by the context-free grammar to qualitatively describe the interactivity between attributes. 2) Through the conversion function, the linguistic expressions are transformed into HFLTSs. The individual evaluation results of all experts are further aggregated with the defined hesitant fuzzy linguistic weighted power average operator (HFLWPA). 3) Based on the standard Euclidean distance formula of the hesitant fuzzy linguistic elements (HFLEs), the hesitant fuzzy linguistic interaction degree (HFLID) between attributes is defined and calculated by constructing a piecewise function. As a result, a 2-order additive fuzzy measure identification method based on HFLID is proposed.

The advantages of this paper may be as follows: 1) The proposed method gives the definition of interactivity between attributes by using the supermodular game theory, which makes the interaction between attributes easy to explain and understand. 2) According to this definition, a linguistic term set is established to characterize the interactivity between attributes. Under the linguistic term set, the proposed method allows the experts to qualitatively describe the interactivity between attributes by using linguistic expressions generated by the context-free grammar, when they are hesitant among multiple possible linguistic information, which reflects both fuzziness and hesitation of decision-making and makes the evaluation of the interactivity between attributes closer to reality. 3) Through the conversion function, the linguistic expressions are transformed into the HFLTSs. The individual evaluation results of all experts are further aggregated by using the defined HFLWPA, which not only considers the weights of experts, but also considers the mutual support degree of opinions of experts, thereby ensuring the rationality of decision-making. 4) The proposed method uses the standard Euclidean distance formula of HFLEs to define and calculate the HFLID between attributes, so the transformation from qualitative description to quantitative characterization is finally realized, thereby better preserving, and characterizing the experts’ evaluation information.

This work proposed a 2-order additive fuzzy measure identification method based on HFLID. Obviously, on the one hand, compared with the objective methods describing and depicting the interactivity between attributes (as in ^7,17–21^ and ^22^), the proposed method has subjectivity. On the other hand, compared with the subjective methods describing and depicting the interactivity between attributes (as in ^9–15^ and ^16^), the proposed method can reflect both fuzziness and hesitation.

By using the HFLTS, this work successfully reflects both fuzziness and hesitation in evaluating the interactivity between attributes in the identification process of 2-order additive fuzzy measure. The proposed method can be widely applied to multi-attribute decision-making problems such as credit evaluation, ESG evaluation, competitiveness evaluation, and technological innovation capability evaluation, which has high practical application value and broad application prospects.

The number of samples and experts used in the application example (see “Application Example”) is small, which weakens the persuasiveness of the analysis results and the stability of the interactivity evaluation. In the future, the number of samples and experts should be increased. Furthermore, the established linguistic term set *S* contains only nine linguistic terms, which is relatively extensive. Therefore, it is necessary to add the linguistic terms in *S* to improve the scientificity of decision-making in the future. At the same time, the credit assessment index system for big data enterprises (see Table 1 in^16^) includes only six primary indicators, it is easier to calculate the 2-order additive fuzzy measure. For example, experts only need to evaluate the interactivity of $C_{6}^{2} = 15$ pairs of attributes and the 2-order additive fuzzy measure only need to determine ${{6 \times (6 + 1)} \mathord{\left/ {\vphantom {{6 \times (6 + 1)} 2}} \right. \kern-0pt} 2} = 21$ parameters. However, when there are many primary indicators, the amount and complexity of calculation will increase significantly. Therefore, it is necessary to design calculation programs to complete calculations in the future. In addition, Method 1 should be compared with other methods except Method 2, Method 3 and Method 4, such as the proportional scaling method^11^, multicriteria correlation preference information method^12^, qualitative cross-impact analysis method^15^, etc. This is one of the shortcomings of this work and needs to be improved in the future.

## Data Availability

Most of the data generated or analyzed during this study are included in the manuscript, and the rest data are promptly available to readers without undue qualifications. The datasets used and/or analyzed during the current study are available from the corresponding author on reasonable request.

## Appendix A

In “Comparative analysis”, the discrimination of the MADM method for alternatives was calculated by using the algorithm of discrimination^42^. Its calculation principle is as follows:

Suppose a decision model or algorithm evaluate the alternatives with decision coefficient *α*, the decision coefficient for alternative $A_{i}$ is $\alpha_{i}$, the decision coefficient for alternative $A_{j}$ is $\alpha_{j}$, and $\alpha_{i} > \alpha_{j}$, then the discrimination of the decision model or algorithm for alternatives $A_{i}$ and $A_{j}$ is defined as

$$\rho_{ij} = \frac{{\alpha_{i} - \alpha_{j} }}{{\alpha_{i} }} \times 100\%$$

Obviously, the larger the discrimination $\rho_{ij}$ is, the better the decision-making effect of the decision model or algorithm for alternatives $A_{i}$ and $A_{j}$ is.
